# Screening Mutations of *MYBPC3* in 114 Unrelated Patients with Hypertrophic Cardiomyopathy by Targeted Capture and Next-generation Sequencing

**DOI:** 10.1038/srep11411

**Published:** 2015-06-19

**Authors:** Xuxia Liu, Tengyong Jiang, Chunmei Piao, Xiaoyan Li, Jun Guo, Shuai Zheng, Xiaoping Zhang, Tao Cai, Jie Du

**Affiliations:** 1 Beijing Anzhen Hospital, Capital Medical University, Beijing, China; 2 Beijing Collaborative Innovation Center for Cardiovascular Disorders; 3 The Key Laboratory of Remodeling-Related Cardiovascular Diseases, Capital Medical University, Ministry of Education, Beijing Institute of Heart, Lung and Blood Vessel Diseases, Beijing 100029, China

## Abstract

Hypertrophic cardiomyopathy (HCM) is a major cause of sudden cardiac death. Mutations in the *MYBPC3* gene represent the cause of HCM in ~35% of patients with HCM. However, genetic testing in clinic setting has been limited due to the cost and relatively time-consuming by Sanger sequencing. Here, we developed a HCM Molecular Diagnostic Kit enabling ultra-low-cost targeted gene resequencing in a large cohort and investigated the mutation spectrum of *MYBPC3*. In a cohort of 114 patients with HCM, a total of 20 different mutations (8 novel and 12 known mutations) of *MYBPC3* were identified from 25 patients (21.9%). We demonstrated that the power of targeted resequencing in a cohort of HCM patients, and found that *MYBPC3* is a common HCM-causing gene in Chinese patients. Phenotype-genotype analyses showed that the patients with double mutations (n = 2) or premature termination codon mutations (n = 12) showed more severe manifestations, compared with patients with missense mutations (n = 11). Particularly, we identified a recurrent truncation mutation (p.Y842X) in four unrelated cases (4/25, 16%), who showed severe phenotypes, and suggest that the p.Y842X is a frequent mutation in Chinese HCM patients with severe phenotypes.

Hypertrophic cardiomyopathy (HCM, MIM# 192600) is defined by an increased left ventricular wall thickness in the absence of other loading conditions^1^. HCM is a common inherited cardiac disorder with an estimated prevalence of 1:500 and often follows an autosomal-dominant inheritance pattern with incomplete penetrance^1^,^2^,^3^. The clinical course of HCM varies considerably, from asymptomatic over limited symptoms to severe heart failure and sudden cardiac death (SCD). Also, HCM is the most common cause of SCD in young and competitive athletes^1^,^4^,^5^. Therefore, early diagnosis is crucial for prevention of such catastrophic events.

Over 1,400 mutations in at least 11 genes encoding sarcomere proteins have been reported to cause HCM^6^. Among them, mutations in the *MYBPC3* gene encoding the myosin binding protein C represent one of the most common genetic causes of HCM^7^,^8^,^9^. It was estimated that ~35% of all cases of HCM are due to *MYBPC3* mutations in Western countries^4^,^6^.

Genetic testing is routinely recommended in patients with HCM according to current clinical guidelines^3^,^7^, but its use at clinic has been limited by the cost and time-consuming conventional sequencing technologies. In this study, we developed targeted exome capture for rapid sequencing and detection of *MYBPC3* mutations. This technology can analyze large genomic regions at lower cost and faster time than conventional Sanger sequencing. It is also regarded as the most advantageous technology in finding almost all types of mutations including small indel mutations^10^,^11^,^12^. Here we report the mutation spectrum of *MYBPC3* in a large cohort of unrelated Chinese patients with HCM using this approach, and explore the clinical characteristics and their correlation with different *MYBPC3* genotypes.

## Results

### Demographic and clinical characteristics

A total of 114 unrelated patients with HCM were recruited from 2012 to 2013. Detailed demographic and clinical characteristics of the patients are summarized in Table 1. Particularly, 29 patients (25.4%) had familial history of HCM, and 12 patients (10.5%) had a family history of unexplained SCD. None of these patients had history of genetic counseling or gene diagnosis.

### Sequence analysis

**Figure 1:**
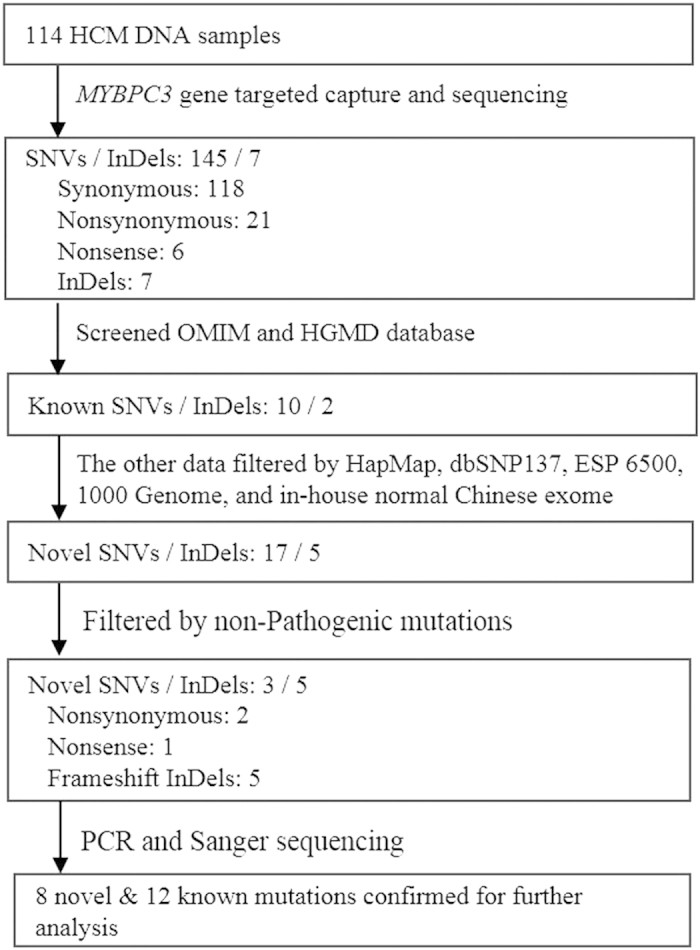
Schematic representation of the filtering process of targeted capture and sequencing data from 114 HCM patients. After known SNVs/InDels were screened, a total of 145 SNVs/ 7 InDels are identified for pathogenic and functional analysis. A total of 20 mutations have been confirmed for phenotype-genotype analysis. SNV, single nucleotide variant; OMIM, Online Mendelian Inheritance in Man; HGMD, human gene mutation database.

**Figure 2:**
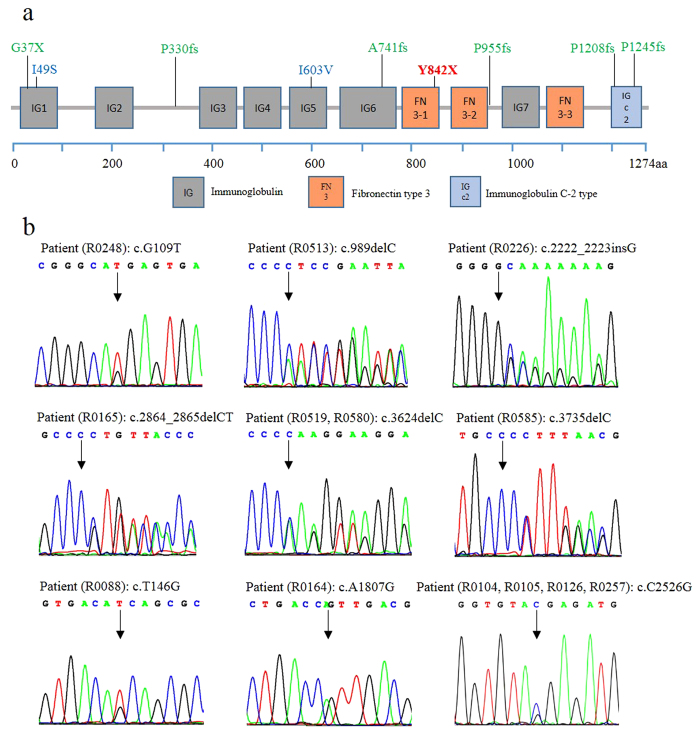
Location and Sanger sequencing of selected mutations in *MYBPC3*. (**a**) *MYBPC3* encodes seven immunoglobulin (IG) domains, three fibronectin type 3 (FN3) domains, and an immunoglobulin C-2 (IGc2) domain. Mutations shown in green indicate seven novel nonsense and frame shift mutations identified in this study. Two novel missense mutations are denoted in blue. The recurrent p.Y842X mutation is presented in red. (**b**) Sanger sequencing has confirmed all 8 novel mutations (2 missense, 1 nonsense and 5 frame shifts) and the p.Y842X mutation. All other 12 known pathogenic mutations also have been confirmed (figure not shown).

Overall, the mean read depth for the exome sequence of *MYBPC3* was >300×. For each subject, more than 78% of the targeted bases were sequenced at least 20 times. The filtering process of targeted capture and sequencing data from 114 HCM patients were shown in Fig. 1. A total of 145 potential mutations (e.g., nonsynonymous, nonsense, and splice-site mutations) and 7 InDels were identified in the 114 patients. By filtering multiple databases (dbSNP137, HapMap, 1000 Genome, ESP6500, and 300 Chinese Han exome in-house database), we identified 8 novel mutations (2 missense, 1 nonsense, and 5 InDel-induced frameshift mutations) as well as 12 known causative mutations (as shown in the HGMD database). All of these 20 mutations were predicted to be probably pathogenic by SIFT and Polyphen-2, and further confirmed by Sanger sequencing analyses (the forward sequencing data are shown in Fig. 2b; the reverse sequencing results are included in Supplementary Fig. S2 online).

### *MYBPC3* genotype analysis

Mutations in the 25 patients (6 with familiar history, 19 sporadic cases) were identified with 20 different mutations in *MYBPC3* (Table 2), including 3 nonsense, 6 InDels, and 11 missense mutations. Here we show the 8 novel mutations and one recurrent mutation on the schematic protein structure (Fig. 2a) and the confirmation of the mutations by Sanger sequencing (Fig. 2b). Four mutations (i.e., p.G37X, p.I49S, p.I603V and p.A741fs) were located in the immunoglobulin (Ig) domains, two mutations (p.P1208fs and p.P1245fs) located in the Ig C2 domain, and only one mutation (p.Y842X) located in the FN3 domain. All of the amino acid residues that are mutated are highly conserved across multiple species from zebrafish to human (Supplementary Fig. S1).

### HCM phenotype associated with *MYBPC3* genotype

Clinical characteristics and corresponding *MYBPC3* mutations of the 25 HCM patients were summarized (Table 3). In the 25 patients, 10 (40%) showed symptoms before 35 years old. Syncope was present in 7 (28%) patients. Six (24%) patients had familial HCM or SCD history. Over half of the patients (64%) developed left ventricular outflow track obstruction (LVOTO).

We further analyzed the correlation of the phenotype-genotype in the patients. Among the 12 patients carrying premature stop codon (PTC) mutations, two patients (R0513 and R0585) carrying novel frame shift mutations (p.P330fs and p.P1245fs) had familial SCD history, severe ventricular arrhythmias or history of receiving septum myectomy due to severe hypertrophy (Table 3). Of note, the p.Y842X, producing a PTC in exon 24, was identified in four unrelated patients showing severe manifestations of hypertrophy requiring surgery or symptomatic arrhythmia. In addition, one patient with the p.S139X mutation was hospitalized for receiving implantable cardioverter-defibrillator.

Two patients with severe manifestations of the disease were detected carrying double mutations (p.G37X and p.R160W; p.I603V and p.R810H). Notably, the p.G37X mutation is the closest premature stop mutation to the starting codon identified thus far, which is predicted to produce a full-truncated protein. And yet, its combination with p.R160W in the patient R0248 may have led to the onset of the disease at younger age, severe clinical symptoms as well as severe hypertrophy for receiving septum myectomy. It was not determined whether these double mutations were compound mutations or simply occurred in the same allele (*cis*). PCR and Sanger sequencing were performed on the available DNA samples from patient (R0164)’s mother and child. The fact that no mutations (p.I603V and p.R810H) were detected in the family members (Supplementary Fig. S3) suggests that the double mutations were derived, as single allele, from the deceased father. However, the possibility of a *de novo* mutation that may occur in either allele in germline cells of fertilized egg or somatic cells of embryonic tissues could not be excluded.

Together, our results have shown that the patients with double mutations (n = 2) or PTC mutations (n = 12) are correlated with more severe manifestations requiring invasive therapies, compared with patients with missense mutations (n = 11) (*p* = 0.01). However, no correlation was found between other clinical indications and missense mutation group, PTC group and double mutations group.

## Discussion

This study for the first time provided exome sequence analysis of *MYBPC3* in Chinese patients by targeted capture and next-generation sequencing. Mutational screening yielded a genetic diagnosis of 25 patients (21.9%) in 114 unrelated HCM patients, suggesting *MYBPC3* is the predominant HCM-causing gene in Chinese cases. The prevalence is similar to the Finnish patients^13^, but higher than that of 12.3% in Denmark^14^ or 15% in European^15^ HCM cases with *MYBPC3* mutations.

Although the spectrum of clinical phenotypes was broad, severe manifestations that required invasive therapies were more frequently found in our patients with disruptive mutations or double mutations, compared to the missense mutations. This finding is consistent with that in 110 consecutive, unrelated patients of European descent^15^. However, Nonsense mutations of *MYBPC3* were also found to be associated with relatively benign clinical course in Japanese and French families^16^,^17^,^18^, suggesting the existing of genetic modifiers for HCM.

It is worth mentioning that among all mutations, the p.Y842X mutation was identified in 4 patients, indicating it is a frequent mutation in Chinese cases. Remarkably, all the patients with p.Y842X showed severe phenotype and ventricular arrhythmias, and mostly requiring invasive therapies. The p.Y842X was also observed in another Chinese pedigree, leading to severe hypertrophy and diastolic dysfunction^19^. In Caucasian, however, only one HCM case was previously reported with p.Y842X^14^. Nevertheless, our data for the first time showed strong association between p.Y842X mutation and severe phenotype in this cohort, indicating this nonsense mutation may be used as a predictor for earlier clinical intervention.

In summary, we have successfully applied the targeted capture and next-generation sequencing technique as a diagnostic tool and identified multiple novel mutations in *MYBPC3*, including the recurrent Y842X mutations, in Chinese patients with HCM. The detection of *MYBPC3* mutation, especially the PTC mutation and double-mutation, may serve as a molecular marker for clinical risk stratification of HCM.

## Methods

### Patient selection

114 unrelated Han Chinese patients, diagnosed as HCM, were consecutively recruited at Anzhen hospital, Capital Medical University, Beijing. Clinically, typical HCM was determined by maximal left ventricular wall thickness (LVWT) ≥15 mm in echocardiography, while atypical HCM was considered by the wall thickness of 13 to 14 mm in the presence of other compelling information (e.g., family history of HCM)^1^. Other loading conditions such as hypertension or aortic valve stenosis were excluded during the screening. Experimental protocol was approved by the Ethics Committee of the Anzhen hospital, and was carried out in accordance with the approved guidelines. Informed consent for genetic analysis was obtained from each of the patients.

### DNA library preparation

Genomic DNAs (gDNAs) were extracted from blood samples using QIAampBlood Midi Kit (QIAGEN, Valencia, CA). Each DNA sample was quantified by agarose gel electrophoresis and Nanodrop (Thermo Scientific, Rockford, IL). DNA libraries were prepared according to Illumina’s protocol. Briefly, 3 μg of gDNAs were fragmented by nebulization; an “A” was then ligated to the 3′-end of the fragmented DNA. The sample was size-selected aiming for a 350 ~ 400 base pair for PCR amplification with a unique index. The final product was validated using the Agilent Bioanalyzer (Agilent, Santa Clara, CA).

### Targeted genes enrichment and sequencing

The amplified DNAs were captured using *MYBPC3* Gene Panel with biotinylated oligo-probes (MyGenostics GenCap Enrichment technologies, Beijing, China). The enrichment libraries were sequenced by Illumina HiSeq 2000 sequencer (Illumina, San Diego, CA).

### Bioinformatics analysis

High-quality reads were retrieved by filtering low quality reads and adaptor sequences using the Solexa QA package and the cut adapt program (http://code.google.com/p/cutadapt/), respectively. SOAP aligner program was used to align the clean read sequences to the human reference genome (hg19). After PCR duplicates were removed by the Picard software, SNPs was firstly identified using the SOAPsnp program (http://soap.genomics.org.cn/soapsnp.html). Subsequently, the selected reads were realigned to the reference genome using BWA. Insertions and deletions (InDels) were identified using the GATK program (http://www.broadinstitute.org/gsa/wiki/index.php/Home_Page). Identified SNPs and InDels were annotated using the Exome-assistant program (http://122.228.158.106/exomeassistant) according to the reference sequence of *MYBPC3* (NM_000256). MagicViewer was used to view short read alignment and validate candidate SNPs and InDels. SNPs and InDels presented in dbSNP137 (http://www.ncbi.nlm.nih.gov/projects/SNP/), HapMap samples, 1000 Genome (http://www.ncbi.nlm.nih.gov/Ftp), and ESP6500 (http://evs.gs.washington.edu/EVS/) were removed. Nonsynonymous variants were evaluated by SIFT (http://sift.bii.a-star.edu.sg/) and Ployphen-2 (http://genetics.bwh.harvard.edu/pph2/) programs to predict their pathogenicity. Sanger sequencing with specific primers (Supplementary Table S1) was applied to confirm the identified mutations^20^.

## Additional Information

**How to cite this article**: Liu, X. *et al.* Screening Mutations of *MYBPC3* in 114 Unrelated Patients with Hypertrophic Cardiomyopathy by Targeted Capture and Next-generation Sequencing. *Sci. Rep.* **5**, 11411; doi: 10.1038/srep11411 (2015).

## Supplementary Material

Supplementary Information

## Figures and Tables

**Table 1:** Clinical findings of 114 patients with HCM.

Examinations	Results
Age at diagnosis or screening (years)	42.9 ± 15.5
Male/Female	72/42
Family history of HCM (%)	
HCM (%)	25.4%
SCD (%)	10.5%
Echocardiography	
Maximal LVWT (mm)	20.8 ± 5.0
Left atrial diameter (mm)	40.6 ± 8.6
LVEDD (mm)	43.7 ± 6.1
LVESD (mm)	27.0 ± 6.2
LVEF	(67.5 ± 9.2)%
Patients with LVOTO (%)	57.9%

Data are presented as the number (%) of subjects or mean value ± SD. HCM, hypertrophic cardiomyopathy; SCD, sudden cardiac death; LVWT, left ventricular wall thickness; LVESD, left ventricular end-systolic diameter; LVEDD, left ventricular end-diastolic diameter; LVEF, left ventricular ejection fraction; LVOTO, left ventricular outflow track obstruction.

**Table 2:** Summary of detected mutations in *MYBPC3*by deeper sequencing.

Patient	Exon	DNA Seq	Amino acid	Mutation type	Domain	Reference
R0248	2	c.G109T	p.G37X	nonsense	IG-1	novel
R0579	4	c.C416A	p.S139X	nonsense	between IG-1 & 2	Kassem (2013)^21^
R0513	12	c.989delC	p.P330fs	frameshift	motif	novel
R0235	15	c.1377delC	p.P459fs	frameshift	IG-4	Lin (2010)^22^; Zou (2013)^23^
R0226	22	c.2222_2223insG	p.A741fs	frameshift	IG-6	novel
R0104, R0105, R0126, R0257	24	c.C2526G	p.Y842X	nonsense	FN3-1	Andersen (2004)^14^
R0165	26	c.2864_2865delCT	p.P955fs	frameshift	motif	novel
R0519, R0580	31	c.3624delC	p.P1208fs	frameshift	IGc2	novel
R0585	32	c.3735delC	p.P1245fs	frameshift	IGc2	novel
R0088	2	c.T146G	p.I49S	missense	IG-1	novel
R0248	4	c.C478T	p.R160W	missense	motif	Anan (2007)^16^
R0598	12	c.G1000A	p.E334K	missense	motif	Anan (2007)^16^
R0168	14	c.G1321A	p.E441K	missense	IG-3	Olivotto (2008)^24^
R0094	16	c.C1504G	p.R502G	missense	IG-4	Richard (2003)^25^
R0103	16	c.G1505A	p.R502Q	missense	IG-4	Niimura (1998)^18^
R0166, R0123, R0227, R0517	16	c.G1519A	p.G507R	missense	IG-4	Erdmann (2003)^15^
R0164	18	c.A1807G	p.I603V	missense	IG-5	novel
R0084	22	c.G2308A	p.D770N	missense	motif	Van Driest (2004)^26^
R0164	24	c.G2429A	p.R810H	missense	FN3-1	Nanni (2003)^27^
R0169	27	c.C2992G	p.Q998E	missense	IG-7	Van Driest (2004)^26^

A list of 2 missense, 1 nonsense and 5 indel-induced frameshift variants were not present in dbSNP135, HapMap, 1000 Genome Project, ESP6500, and in-house normal Chinese exome database. Harmful alleles were predicted by both SIFT and PPH2 programs. Amino acid alterations due to splice site mutations have not been determined.

**Table 3:** Analyses of HCM phenotype and *MYBPC3* genotype.

Patient	Mutation	Sex	Age at onset (years)	Duration	Syncope	NYHA class	FHCM or FSCD	LVWT max (mm)	Site	LA (mm)	LVEDD (mm)	LVEF (%)	LVOTO	Invasive therapy	ECG
**Frameshift and/or nonsense**															
R0513	p.P330fs*	M	20	20		4	FSCD	28.4	S	31	40	73	Y	SM	VT, LVH, ST-T *abn.*
R0235	p.P459fs	M	61	3		1		32	S	42	47	63	Y		LVH, ST-T *abn.*
R0226	p.A741fs*	M	21	5		2		24	S	46	40	69	Y		LVH, ST-T *abn.*
R0165	p.P955fs*	F	47	1		1		16	S	28	41	60	Y		VT
R0519	p.P1208fs*	M	51	5		1		16.5	S	41	44	70			ST-T *abn.*
R0580	p.P1208fs*	F	UK	UK		UK		15.8	S	39	45	69		UK	UK
R0585	p.P1245fs*	M	25	1	Y	1	FHCM FSCD	14	S	38	52	65			PVC, ST-T *abn.*
R0579	p.S139X	M	45	16		4		28.5	A	50	41.5	78	Y	ICD	ST-T *abn.*, LVH
R0104	p.Y842X	M	47	10	Y	4		28	A	44	44	75	Y	ASA	ST-T abn., LVH, LAH
R0105	p.Y842X	M	47	11	Y	4		27	S	35	40	61	Y	ASA, PM	1-AV block
R0126	p.Y842X	M	40	34		4	FHCM	19	S	46	57	45		PM	AF
R0257	p.Y842X	F	24	37	Y	4	FHCM	13.9	S	46	51	69			UK
**Mean** ± **SD**			38.9 ± 14.0	13.0 ± 12.7				21.9 ± 6.7		40.5 ± 6.6	45.2 ± 5.5	66.4 ± 8.7			
**Double mutations**															
R0248	p.G37X*; p.R160W	M	25	10		3		26	S	43	41	80		SM	RBBB, LVH, QTP, ST-T *abn.*
R0164	p.I603V*; p.R810H	M	20	20		4		28	S	42	47	82	Y		LVH, ST-T *abn.*
**Mean** ± **SD**			22.5 ± 3.5	15 ± 7.1				27 ± 1.4		42.5 ± 0.7	44 ± 4.2	81 ± 1.4			
**Missense**															
R0088	p.I49S*	F	39	10		4		18	S	45	45	75			MI
R0598	p.E334K	F	37	10		2	FHCM	20	S	43	47	65	Y		LVH, ST-T *abn.*
R0168	p.E441K	M	45	15	Y	3	FHCM	26	S	39	ND	61	Y		IVCB, MI, LVH, ST-T *abn.*
R0094	p.R502G	F	29	14		3		21	S	32	38	66	Y		QTP
R0103	p.R502Q	M	38	4		4		16.4	S	32	43	59			RBBB, LVH, ST-T *abn*.
R0123	p.G507R	F	52	2		2		18	S	31	47	54	Y		LAH
R0166	p.G507R	M	30	26	Y	1		20	S	46	52	55	Y		AF, LVH, ST-T *abn.*
R0227	p.G507R	F	24	1		1		24.5	S	37	38	65	Y		UK
R0517	p.G507R	M	43	1		2		21	S	36	44	69			WPW
R0084	p.D770N	M	48	1	Y	1		17	S	43	40	78	Y		ST-T *abn.*
R0169	p.Q998E	M	29	1		1		13.5	S	36	45	67	Y		LVH, ST-T *abn.*
**Mean** ± **SD**			37.6 ± 8.9	7.7 ± 8.1				19.6 ± 3.6		38.2 ± 5.4	43.9 ± 4.4	64.9 ± 7.5			

^*^novel mutation found in this study. Blank: negative result. Y, yes; UK, unknown; S, septum; A, apex; FHCM, family history of HCM; FSCD, family history of sudden cardiac death; max, maximum; LA, left atrial internal diameter; SM, septum myectomy; RBBB, Right Bundle Branch Block; LVH, left ventricular hypertrophy; QTP, QT duration prolongation; *abn.* abnormalities; ICD, implantable cardioverter-defibrillator; VT, ventricular tachycardia; LAH, left atrial hypertrophy; ASA, alcohol septal ablation; MI, myocardial infarction; PM, pacemaker; 1-AV, first-degree atrioventricular; AF, atrial fibrillation; IVCB, intraventricular conduction block; LVOTD, left ventricular outflow track dredging; WPW, Wolff–Parkinson–White syndrome. ND, not determined. The other abbreviations were seen in the Table 1.
