# Supplementary material for: Screening Mutations of MYBPC3 in 114 Unrelated Patients with Hypertrophic Cardiomyopathy by Targeted Capture and Next-generation Sequencing
Source: Sci Rep. 2015 Jun 19;5:11411. doi: 10.1038/srep11411 (PMC4473690; doi:10.1038/srep11411)
Supplement: Supplementary Information [file srep11411-s1.doc]

**Supplementary information**

**Screening Mutations of *MYBPC3* in 114 Unrelated Patients with Hypertrophic Cardiomyopathy by Targeted Capture and Next-generation Sequencing**

Xuxia Liu^1,2,3^, Tengyong Jiang^1^, Chunmei Piao^1,2,3^, Xiaoyan Li^1,2,3^, Jun Guo^1,2,3^, Shuai Zheng^1,2,3^, Xiaoping Zhang^1,2,3^, Tao Cai^1^, Jie Du^1,2,3,*^

^1^Beijing Anzhen Hospital, Capital Medical University, Beijing, China; ^2^Beijing Collaborative Innovation Center for Cardiovascular Disorders; ^3^The Key Laboratory of Remodeling-Related Cardiovascular Diseases, Capital Medical University, Ministry of Education, Beijing Institute of Heart, Lung and Blood Vessel Diseases, Beijing 100029, China.

*Correspondence: Jie Du ([jdu@bcm.edu](mailto:jdu@bcm.edu))

**Supplementary Figures**


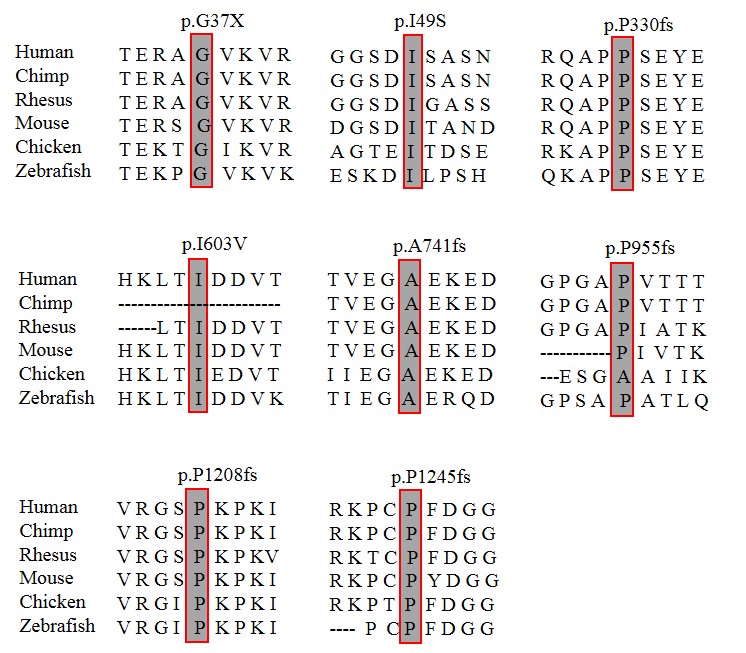


**Figure S1**∣**Multiple sequence alignments.** Multiple sequence alignments for the *MYBPC3* peptide sequences flanking the residues (in red box) that are mutated. All of 8 novel mutational amino acid residues are highly conserved across six species from zebrafish to human.


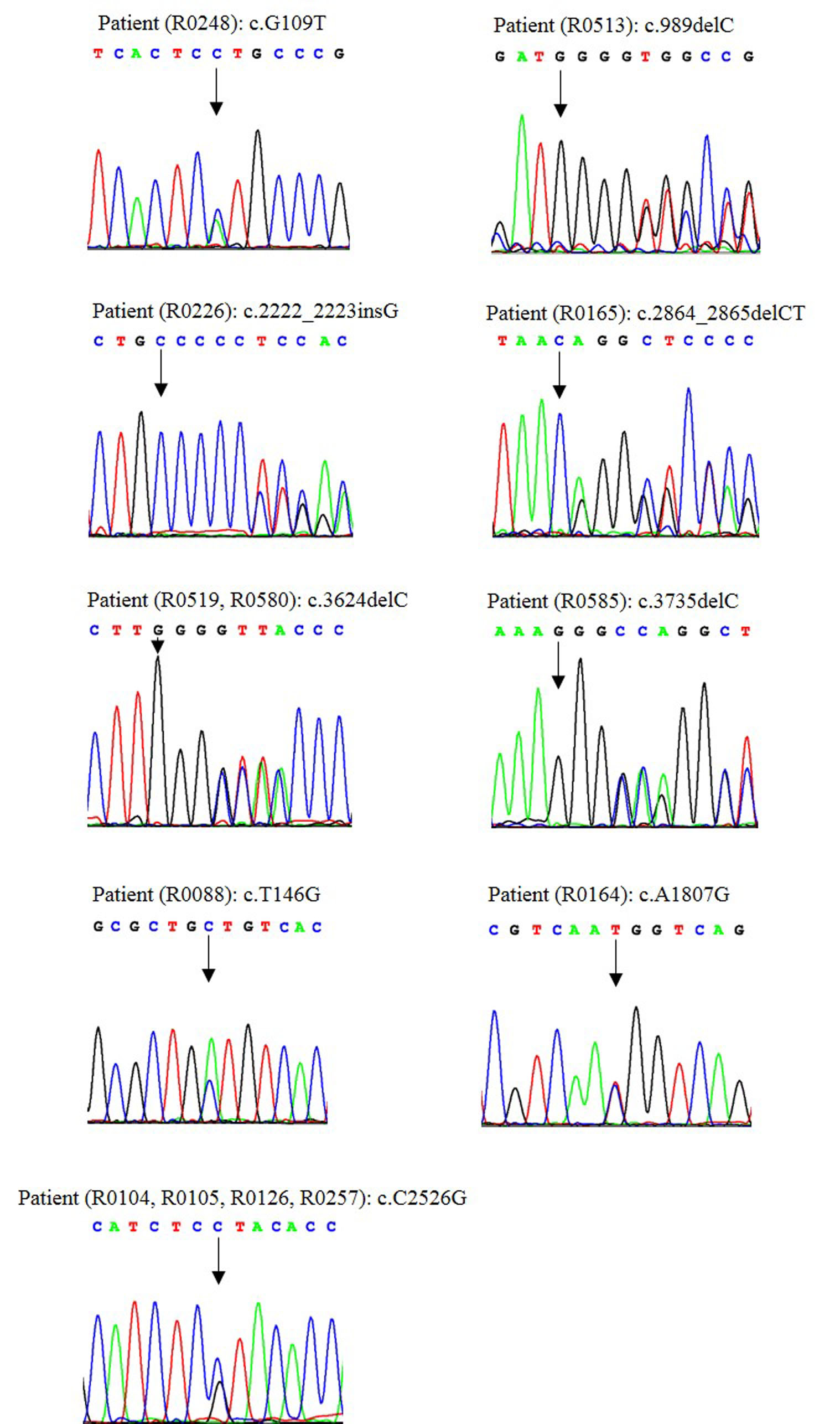


**Figure S2**∣**Reverse sequencing results of the mutations of *MYBPC3.***


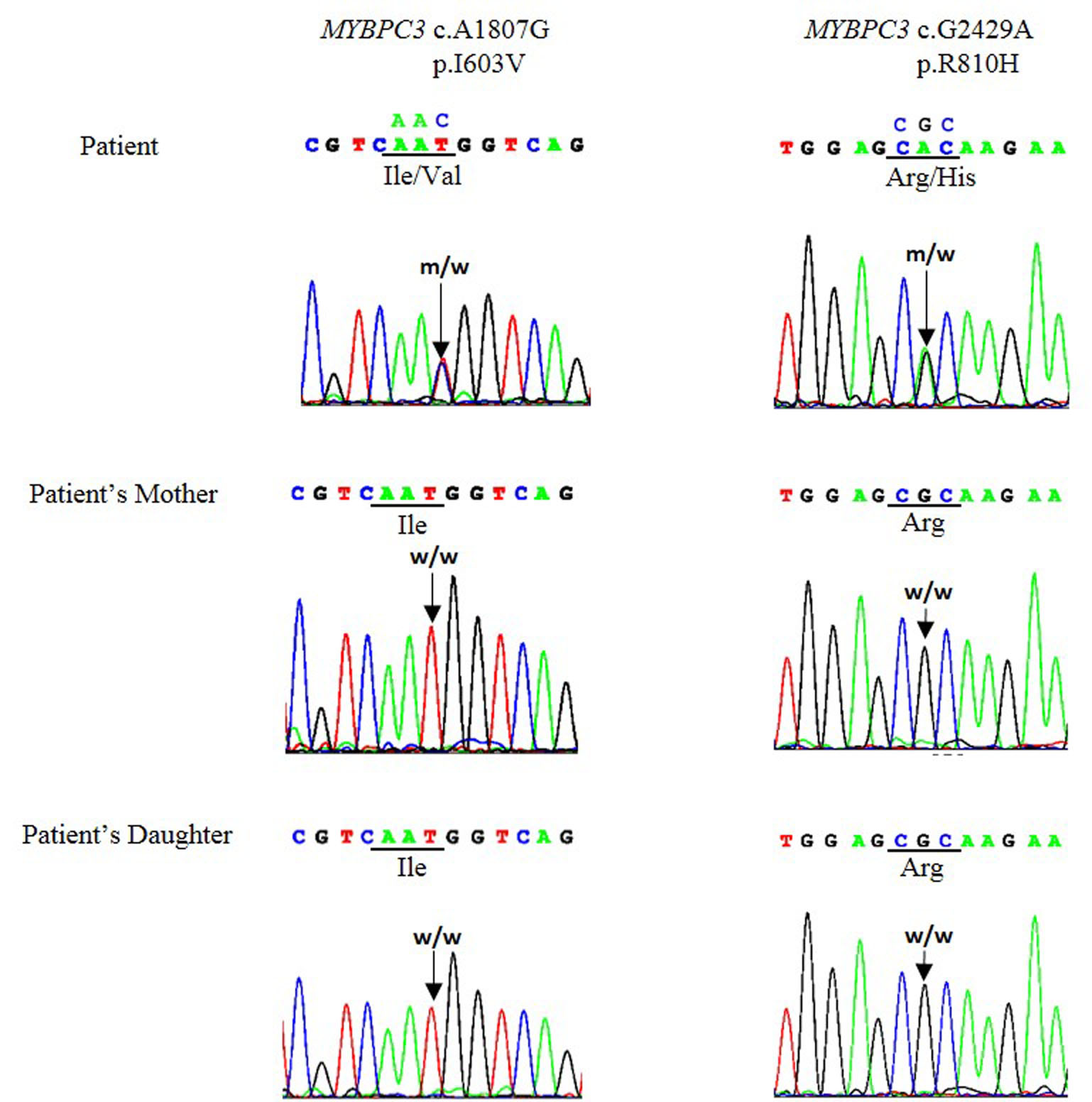


**Figure S3**∣**Sequence analysis of the two mutations (p.I603V and p.R810H) of *MYBPC3* in patient R0164.** The Ile603Val and Arg810His mutations were identified in the patient, but no mutations were found in the patient’s mother and daughter. The fragment from the patient containing the A→G and G→A transversion in position 1807 and 2429 (arrows). “w”, wild-type allele; “m”, mutant allele.

**Supplementary Tables**

**Supplementary Table S1∣Primers used for *MYBPC3* mutation sites Sanger sequencing**

| *MYBPC3* DNA Mutation Site Primers | Forward primer sequence (5'→3') | Reward primer sequence (5'→3') | Product length (bp) |
| --- | --- | --- | --- |
| c.G109T | AGCCTCAGTGTCCTCCTCTG | AAACCTCAGGGAAGGCTGAT | 473 |
| c.T146G | ACGTGAGGGGATGCATAGAA | GTGAAAGCACCTCCTGTTCC | 473 |
| c.C478T | CTGCGGTCCCAGCTAACTT | GGTCGTAGCTGTCGTGCAG | 495 |
| c.A706G | TCCCAAAGTGGGGATTACAG | AACAGAGTCCCACCCAGATG | 465 |
| c.989delC | TCATCACCCCTAATTCTGCC | GGCTAACCTATGCCCTCTCC | 474 |
| c.G1000A | CCCCTCTTCAGCTCCCTTG | CCATGTATGTGGACGAGGTG | 492 |
| c.G1321A | TTTTGTCTCGGGCTCACTTC | CCCTCCTCCGATACTTCACA | 479 |
| c.1377delC | ATCTTTGAGTCCATCGGTGC | TGATCAGGTGGTGTCTCTGC | 480 |
| c.C1504G | AGAGCCCCCTGTGCTCAT | GTCAAAGGCCCAAGGTCAC | 484 |
| c.G1505A | TGTGAAGTATCGGAGGAGGG | CCCTCTCAGTACCCTCTGGA | 484 |
| c.G1519A | TGTGAAGTATCGGAGGAGGG | CCCTCTCAGTACCCTCTGGA | 483 |
| c.A1807G | CTGGGGTATCTGGCAAGG | GCTCTTCCCTCTGTGAGTGG | 399 |
| c.2222_2223insG | CCCGTGACAAAGCTAGAACC | GGCAGAAAAACCTGTCCTGT | 499 |
| c.G2308A | TCTGACTTGGATCTCACCCC | AGCAGTGTCGCAGGAAATCT | 404 |
| c.G2429A | CATCCTGGGTGAGTGCAAG | AGGACCAGGCCAGGCAGGCT | 460 |
| c.C2526G | CATCCTGGGTGAGTGCAAG | CATCTGTAAAATGCGGCTGA | 460 |
| c.2864_2865delCT | GGTGTCAGTGGTGACACAGC | CCACCCTCTCTGCACTTTTT | 401 |
| c.C2992G | CCTTGTCTCAAGGGAGGTTG | CCTGGTAAGTGCCTGAATGC | 480 |
| c.3624delC | ACCCTCATTCTTCCAACCAG | GGACCCAGGGAGACACATC | 439 |
| c.3735delC | ATGTTTGTTTCCAGCCTTGG | TCTGGAAGCTATTGCCCATC | 477 |

**Supplementary Table S2∣Comparison between the reported *MYBPC3* mutations and the present study**

| **Mutation**  **Position** | **Sex** | **Age at Onset (years)** | **Duration** | **FHCM or FSCD** | **LVWTmax (mm)** | **Site** | **LA (mm)** | **LVEDD (mm)** | **LVEF (%)** | **LVOTO** | **Invasive therapy** | **ECG** | **Comments** |
| --- | --- | --- | --- | --- | --- | --- | --- | --- | --- | --- | --- | --- | --- |
| p.P459fs | M | 61 | 3 |  | 32 | S | 42 | 47 | 63 | Y |  | LVH, ST-T *abn.* | this study |
| p.P459fs | F | 59 | 2 | FHCM | 20 | S | 36 | 40 | 78 |  |  | ST *abn.*, Q wave *abn.* | Lin (2010)^22^ |
| p.P459fs | F | 24 | 10 | FHCM | 28 | S | 30 | 40 | 77 |  |  | ST *abn.* | Lin (2010)^22^ |
| p.R160W | M | 69 | 6 |  | 16 | S |  |  |  |  |  | LVH | Anan (2007)^16^ |
| p.G37X,160W | M | 25 | 10 |  | 26 | S | 43 | 41 | 80 |  | SM | RBBB, LVH, QTP, ST-T *abn.* | this study |
| p.R810H | M | 39 |  | FHCM | 32 | S |  | 37 | 60 | Y |  |  | Nanni (2003)^27^ |
| p.R810H,R820Q | M | 53 |  |  | 23 | S |  | 44 | 70 |  |  |  | Nanni (2003)^27^ |
| p.I603V,R810H | M | 20 | 20 |  | 28 | S | 42 | 47 | 82 | Y |  | LVH, ST-T *abn.* | this study |
| p.E334K | F | 37 | 10 | FHCM | 20 | S | 43 | 47 | 65 | Y |  | LVH, ST-T *abn.* | this study |
| p.E334K | M | 60 | 3 |  | 22 | S |  |  |  |  |  | Giant Negative T wave | Anan (2007)^16^ |

Blank: negative result. Y, yes; S, septum; FHCM, family history of hypertrophic cardiomyopathy; FSCD, family history of sudden cardiac death; LVWTmax, maximum left ventricular wall thickness; LA, left atrial internal diameter; LVEDD, left ventricular end-diastolic diameter; LVEF, left ventricular ejection fraction; LVOTO, left ventricular outflow track obstruction; LVH, left ventricular hypertrophy; *abn.*, abnormalities; SM, septum myectomy; RBBB, Right Bundle Branch Block; QTP, QT duration prolongation.
